# Supplementary material for: The Anti-Inflammatory and the Antinociceptive Effects of Mixed Agrimonia pilosa Ledeb. and Salvia miltiorrhiza Bunge Extract
Source: Plants (Basel). 2021 Jun 17;10(6):1234. doi: 10.3390/plants10061234 (PMC8234973; doi:10.3390/plants10061234)
Supplement: Supplementary file 1 [file plants-10-01234-s001.zip › plants-1249190-supplementary.pdf]

Supplementary information

The anti-inflammatory and the antinociceptive effects of mixed *Agrimonia pilosa* Ledeb. and *Salvia miltiorrhiza* Bunge extract

Jinghui Feng[1,2,†], Hyun Yong Kim[3,†], Su Min Sim[1,2], Guanglei Zuo[3], Jeon Sub Jung[2], Seung Hwan Hwang[3,4], Youn Gil Kwak[5], Min Jung Kim[5], Jeong Hun Jo[5], Sung Chan Kim[6], Soon Sung Lim[3,*] and Hong Won Suh[1,2,*]

1 Department of Pharmacology, College of Medicine, Hallym University, 1 Hallymdaehak-gil, Chuncheon, Gangwon-do 24252, Republic of Korea; B17501@hallym.ac.kr (J.H.F.); sumin@hallym.ac.kr (S.M.S.); hwsuh@hallym.ac.kr (H.W.S.)

2 Institute of Natural Medicine, College of Medicine, Hallym University, 1 Hallymdaehak-gil, Chuncheon, Gangwon-do 24252, Republic of Korea; de3180@hallym.ac.kr (J.S.J.)

3 Department of Food Science and Nutrition, College of Natural Science, Hallym University, 1 Hallymdaehak-gil, Chuncheon, Gangwon-do 24252, Republic of Korea; khy9514@nate.com (H.Y.K.); B16504@hallym.ac.kr (G.L.Z.); isohsh@gmail.com (S.H.H.); limss@hallym.ac.kr (S.S.L.)

4 R&D Center, Huons Co., Ltd., 55 Hanyangdaehak-ro, Ansan, Gyeonggi-do 15588, Republic of Korea;

5 Research Institute, Huons Nature, Geumsan, Choong-cheong Nam-do 32742, Republic of Korea; kyg@huonsnature.com (Y.G.K.); jas.mjkim@gmail.com (M.J.K.); jhjo@huonsnature.com (J.H.J.)

6 Department of Biochemistry, College of Medicine, Hallym University, 1 Hallymdaehak-gil, Chuncheon, Gangwon-do 24252, Republic of Korea; biokim@hallym.ac.kr

† The authors contributed to the manuscript in equal measure.

* Co-Correspondence: limss@hallym.ac.kr; Tel.: +82 33 248 2133; and hwsuh@hallym.ac.kr; Tel.: +82-33-248-2614

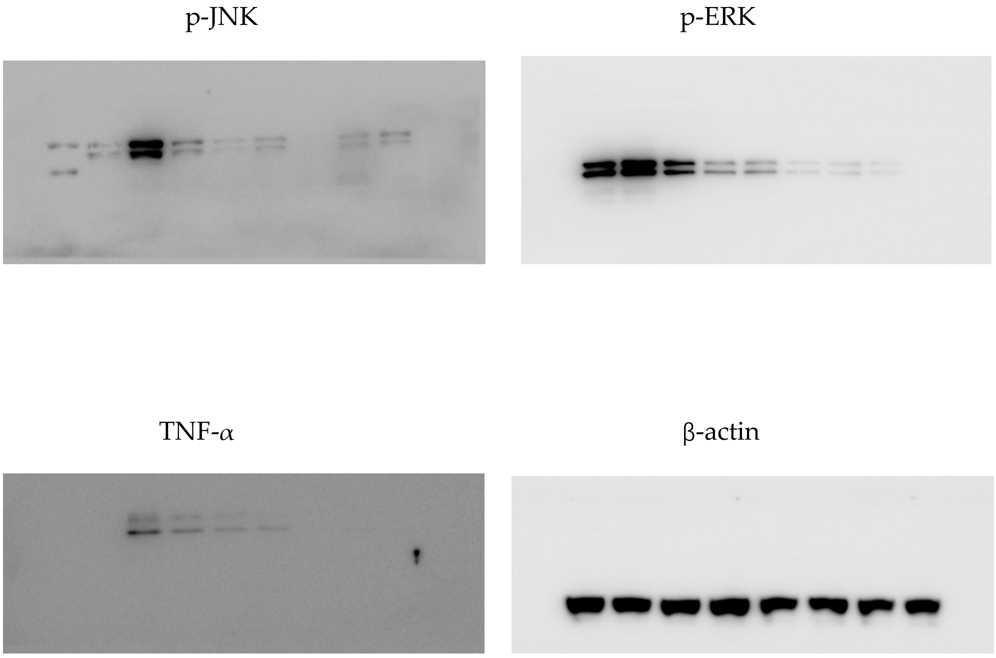

Supplementary Figure 1. Representative full-gel images of Western blots in Figure 5A.

p-JNK

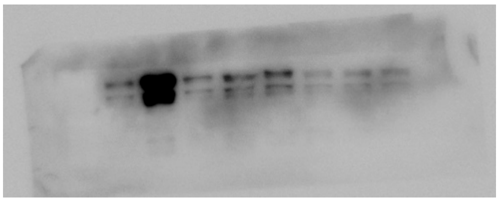

p-ERK

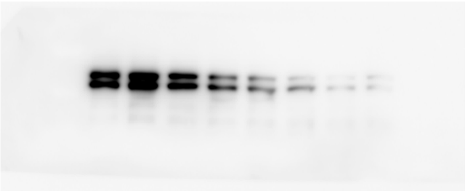

TNF-α

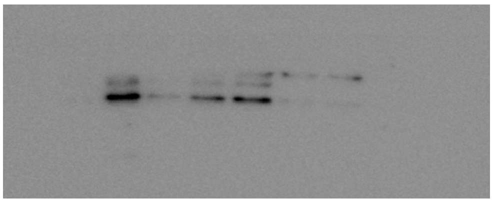

β-actin

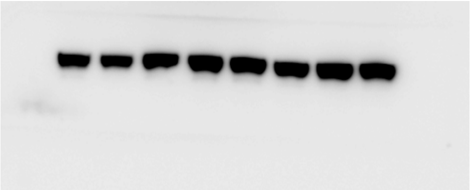

Supplementary Figure 2. Representative full-gel images of Western blots in Figure 6A.

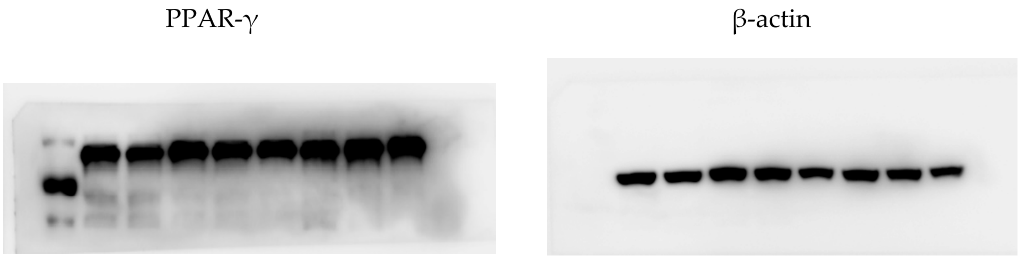

Supplementary Figure 3. Representative full-gel images of Western blots in Figure 7A.

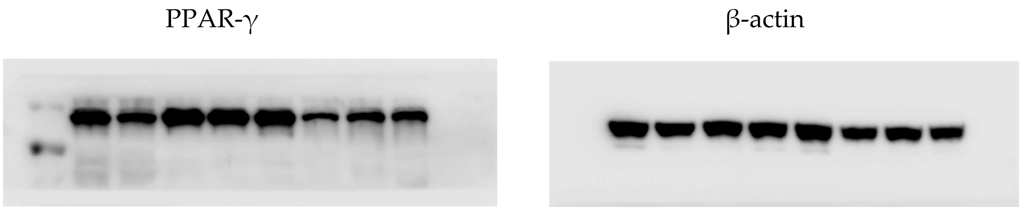

Supplementary Figure 4. Representative full-gel images of Western blots in Figure 7B.
